# Investigating Stoichiometric Controls of Nutrient Recycling in Rivers Using the Threespine Stickleback (*Gasterosteus aculeatus*)

**DOI:** 10.1002/ece3.71920

**Published:** 2025-09-16

**Authors:** Sarah R. Rozanski, Emily M. May, Rana W. El‐Sabaawi

**Affiliations:** ^1^ Department of Biology University of Victoria Victoria British Columbia Canada

**Keywords:** bone, egestion, excretion, nutrient recycling, phenotypic variation, phosphorus

## Abstract

In freshwater ecosystems, fish play a critical role in regulating the standing stock and turnover rates of biologically important elements such as nitrogen (N) and phosphorus (P). They do so by assimilating dietary nutrients, storing them, and recycling a subset as liquid (excreta) or solid (egesta) waste. The elemental composition of fish diets, bodies and waste varies considerably both within and between species. However, the mechanistic link between intraspecific variation in particular traits, such as bone investment, and variation in waste elemental composition and release rates remains poorly understood. Using the framework of Ecological Stoichiometry, we investigate how phenotypic traits impact nutrient storage and recycling. The threespine stickleback serves as an ideal model because it has undergone significant phenotypic diversification following its relatively recent colonisation of freshwater environments. Specifically, we examine variation in P‐rich bony armour along a river continuum, where estuarine sticklebacks are typically heavily armoured (fully plated), and freshwater sticklebacks have less armour (low plated). We collected sticklebacks from five sites along the Sooke River, ranging from the estuary to the upper reaches, and measured their excretion rates in the field. We quantified the P content of their bodies, diet, egesta and excreted wastes, and the N:P of their bodies and excreta. Our results revealed substantial variation in body P content (2.2%–5.9%), with fully plated fish exhibiting higher body %P and lower body N:P. Dietary P was highly variable, with fully plated fish showing marginally higher dietary %P. Notably, P excretion rates were positively correlated with body %P but not diet %P, suggesting that contrary to predictions, bone content may decrease P demand. This study demonstrates that differences in stickleback bone investment have led to meaningful differences in nutrient storage and recycling.

## Graphical abstract

We determine how intraspecific phenotypic variation relates to controls on vertebrate nutrient cycling. We used the threespine stickleback as a model, and ecological stoichiometry as a framework. We found a large portion of variation in nitrogen and phosphorus content of stickleback bodies and wastes, and we find a link between vertebrate traits and excretion that could be explained by variation in elemental homeostasis.

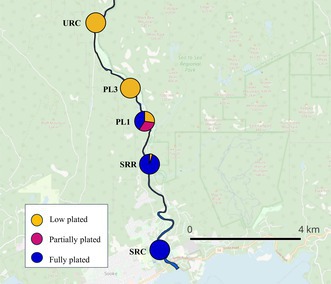

## 1 Introduction

Vertebrates are incredibly important in nutrient cycles because they are relatively long lived, large, nutrient‐rich, and have high excretion rates (Doughty et al. 2016; McIntyre et al. 2007; Vanni and McIntyre 2016; Wenger et al. 2019). Among vertebrates, fishes can substantially impact nutrient cycling (McIntyre et al. 2008; Vanni and Layne 1997). Fish can directly contribute to nutrient cycles by providing a relatively steady supply of biologically essential elements, such as nitrogen (N) and phosphorus (P), to primary producers and microbes via their excretion, which keeps production stable in the face of inconsistent external nutrient inputs (Atkinson et al. 2016; Brabrand et al. 1990). Fish vary dramatically in their excretion rates (Allgeier et al. 2014; McIntyre et al. 2008; Sereda et al. 2008; Vanni and McIntyre 2016). They also vary, both intra‐ and interspecifically, in many other phenotypic traits that can alter excretion, such as body size (Sereda et al. 2008; Vanni and McIntyre 2016). Given the importance of fish in nutrient cycling, it is critical that we understand how trait variation drives excretion rates.

Ecological stoichiometry (ES) is a framework that expresses animal bodies, diets, and wastes as elemental ratios (Sterner and Elser 2002). The proportion of elements in animal bodies can give us insight into the proportions of tissues and biomolecules: Carbon (C) is correlated with carbohydrates and lipids, N with muscle (proteins) and nucleic acids, and P with nucleic acids and bone (Hendrixson et al. 2007; Sterner and Elser 2002). For vertebrates, the majority of P (~80%) is found in bone (Gillooly et al. 2005; Pasteris et al. 2008). ES assumes that the elemental compositions of animal bodies (their organismal stoichiometry, OS) are homeostatic and are maintained by mass balance between diet and waste (Hall 2009; Sterner and Elser 2002). Therefore, OS should reflect the dietary demand, and the composition of the waste should reflect the mismatch between OS and the diet (Elser and Urabe 1999). However, many studies do not fully support these predictions (Vanni and McIntyre 2016), especially in vertebrates (El‐Sabaawi et al. 2016; Sterrett et al. 2015). This could be because animals can meet their elemental demand through a variety of mechanisms, such as selective feeding or increased assimilation efficiency, which may mask altered excretion if researchers do not analyse diet stoichiometry (Vanni and McIntyre 2016). Alternatively, vertebrates may not be as homeostatic as previously assumed (May and El‐Sabaawi 2024). Specifically, the vertebrate bone hypothesis (VBH) posits that bone is a metabolically active tissue that can store and release P to meet current demands (May and El‐Sabaawi 2024).

The majority of studies concerning the stoichiometry of vertebrates focus on interspecific variation (Benstead et al. 2014; McIntyre and Flecker 2010). However, it is also important to understand intraspecific variation in stoichiometry, because it can be substantial and can affect ecological processes (Bassar et al. 2010; Harmon et al. 2009; Rudman et al. 2015). The effect of intraspecific variation, and specifically variation in OS, on ecological processes has only recently been appreciated, and the drivers of this variation remain uncertain (Durston and El‐Sabaawi 2017; El‐Sabaawi et al. 2016, 2015; Leal et al. 2017; Moody et al. 2018; Wei et al. 2022). ES studies have shown that morphological traits affect OS and explain some of its variation (Durston and El‐Sabaawi 2017; Lemmen et al. 2019; Sterrett et al. 2015). However, few studies use the OS of a single vertebrate species to test ES predictions about excretion, and those that have often found counterintuitive results (El‐Sabaawi et al. 2016; Durston and El‐Sabaawi 2019).

**FIGURE 1:**
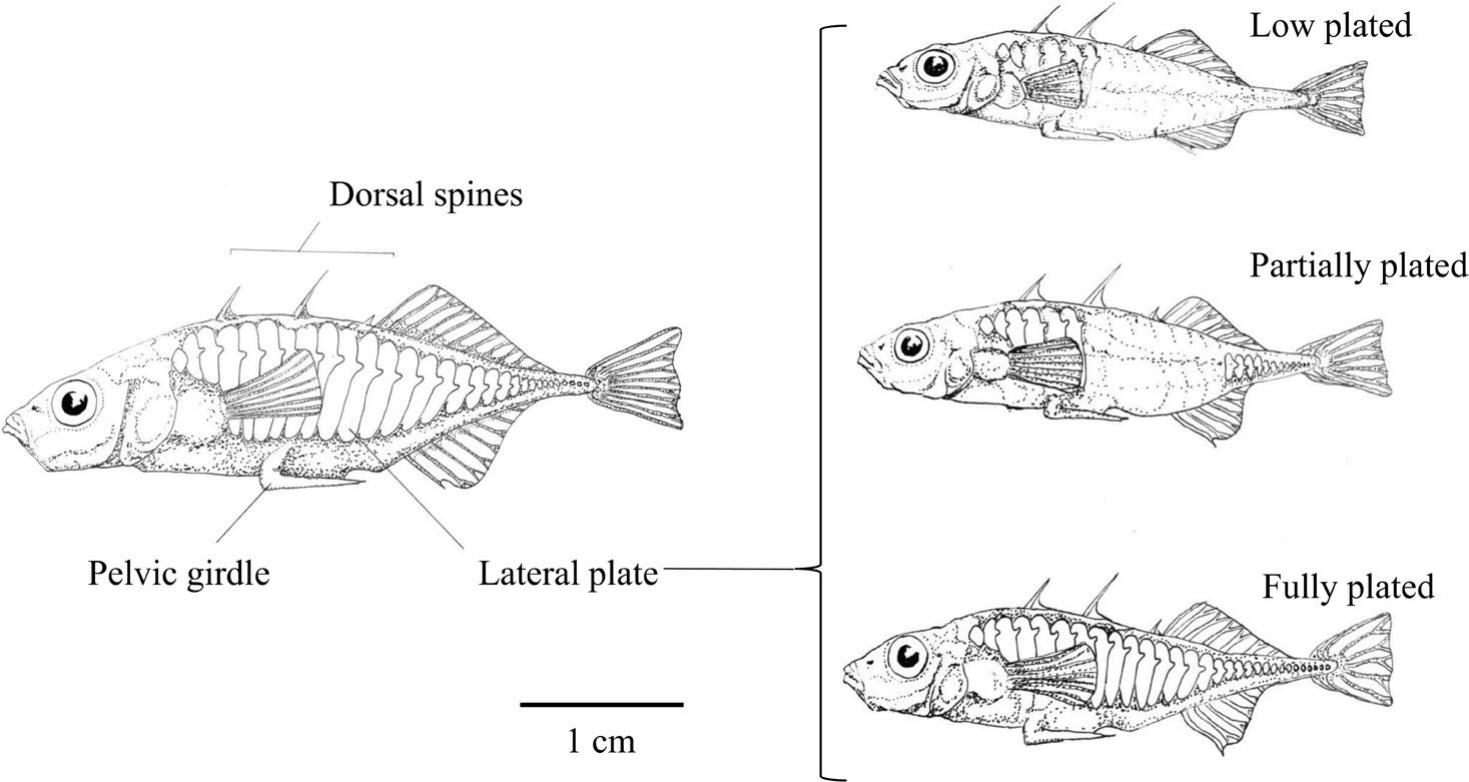
The primary armour traits of the threespine stickleback: Dorsal spines, pelvic girdle, and lateral plates, with the three plate morphs common to threespine sticklebacks shown on the right: Low plated (top), partially plated (middle) and fully plated (bottom). Sticklebacks drawn by Sarah Rozanski.

*Gasterosteus aculeatus* L. (hereafter the threespine stickleback) is a species of ray‐finned fish that has well‐documented variation in its OS stemming in part from its variation in bony armour (Archambeault et al. 2020; Bell and Foster 1994; Durston and El‐Sabaawi 2017; Leal et al. 2017). This variation is largely a result of adaptive radiation since sticklebacks' relatively recent colonisation of freshwater environments around 12,000 years ago in North America (Schluter 1993; Thompson et al. 1997). Among other phenotypic differences, this has resulted in differences in P‐rich bony armour investment (Hagen and Gilbertson 1972). This armour includes lateral plates, dorsal and pelvic spines, and a pelvic girdle (Figure 1). Within a river system, there is often a transition between marine populations, which are almost exclusively fully plated and considered phenotypically similar to the ancestral form, and more derived freshwater populations, which are generally low plated (Bell and Foster 1994; Hagen and Gilbertson 1972). Hybrids can be fully or partially plated due to the partial dominance of alleles at the *Eda* locus (Aguirre and Bell 2012; Colosimo et al. 2005) and are found in ‘hybridisation zones’ at a certain point in the landscape continuum (Thompson et al. 1997; Schluter 2000). The reduction of lateral plates is associated with a significantly lower body P content and a higher body N:P (Durston and El‐Sabaawi 2017). Therefore, armour diversification in threespine sticklebacks has changed how N and P are stored and potentially cycled. Experimental evidence shows that intraspecific variation in threespine sticklebacks can affect community composition, primary productivity, and water quality (Harmon et al. 2009). Currently, however, the mechanisms by which sticklebacks' variation causes ecosystem change are unknown (El‐Sabaawi et al. 2016; Rudman et al. 2019, 2015). River systems are a unique frontier within which to study intraspecific variation because we can elucidate the ecological consequences of divergent selective pressures on a single species.

We aim to better understand factors influencing vertebrate excretion using ES. Specifically, we explore intraspecific variation in morphology (e.g., lateral plate morph, gut length, size, body condition), OS (%P, %N, N:P), excretion stoichiometry, and diet and egesta stoichiometry. We predict that OS will vary based on lateral plate morphology because bone is a very P‐rich tissue. We predict that body %P will be greater in fully plated fish because they have relatively more bone. Because a greater gut length is often correlated with greater nutrient absorption (Karasov and Douglas 2013), we predict that gut length will be negatively correlated with P content of egesta. Because excretion rates can be mediated by body size (Sereda and Hudson 2011; Vanni and McIntyre 2016), we predict that P excretion rate will be positively related to standard length. We will assess how excretion stoichiometry relates to diet and OS.

We have three competing predictions for the relationship between diet, body, and waste stoichiometry. The first, based on classic ES predictions, is that P excretion rate may be lower in fish with higher body P because they meet their relatively higher demand for P by increasing the relative assimilation of P in the tissues, leading to a negative relationship between body and excretion P. If this is the case, we also predict that fish investing more heavily in bone (fully plated morphs) might have a higher absorption of P in the gut compared to low plated morphs. The second is that sticklebacks with higher body P will selectively feed on P‐rich prey, so diet P will be positively related to body P, and P excretion rate will not have any relationship with body P. The third prediction is based on the VBH (May and El‐Sabaawi 2024), which postulates P demand can decrease with increasing bone content, as a result of bone's role as a P reservoir. Therefore, we predict that there will be a positive correlation between body P and excretion.

## 2 Methods

### 2.1 Study Area

The Sooke River watershed is situated on the southern end of Vancouver Island in the Coastal Douglas Fir biogeoclimatic zone (Barlak 2019). The Sooke watershed has been inhabited by the T'Sou‐ke First Nation for thousands of years, and the relationship between the people and the land continues to this day (T'Sou‐ke Nation 2021). Sticklebacks are plentiful in the Sooke River estuary and are, in fact, the namesake of the T'Sou‐ke Nation and the greater Sooke area (T'Sou‐ke Nation 2021). Much of the Sooke watershed (~45%) has been logged, and today the forests are primarily second growth (CRD 2017). While some land immediately surrounding the Sooke River is urbanised, the provincial and regional parks, as well as the T'Sou‐ke Nation, protect much of the surrounding area (CRD 2017; T'Sou‐ke Nation 2021).

The Sooke River watershed is an exorheic system that drains a total area of 340 km^2^ (Barlak 2019). The Sooke River is approximately 20 km long, with a maximum depth of 6.5 m (Barlak 2019). Its flow regime is dominated by rainfall, and its mean annual discharge is 9.5 m^3^/s (Burke 2002). There is a canyon section approximately 7 km from the mouth of the river that is considered impassable to anadromous species because it has 4 separate waterfalls with drops of 4–6 m (Pendray 1980). To our knowledge, all previous field assessments of the sticklebacks in Sooke have sampled only from the estuary and have excluded any other freshwater river sites (Durston 2016; Durston and El‐Sabaawi 2019, 2017; May and El‐Sabaawi 2022a, 2022b).

### 2.2 Field Sampling and Incubations

**FIGURE 2:**
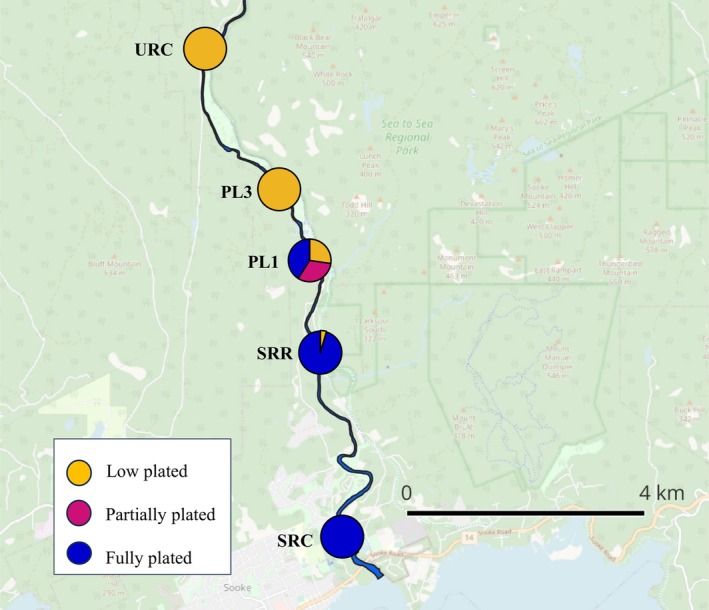
A map of the Sooke River with the five sampling sites indicated. The pie charts represent the plate morphs (low, partially, or fully plated) present at each of the five sampling sites: The Sooke River Campsite (SRC), Sooke River Road (SRR), Parking Lot 1 (PL1), Parking Lot 3 (PL3), and the Upper River Campsite (URC).

Threespine sticklebacks were collected from five sites along the Sooke River from the estuary to 10.6 km upriver (Figure 2 and Table S1). Sampling took place between late June and early July, while sticklebacks were reproducing. Gee minnow traps were baited with inaccessible cat treats and allowed to soak for 1–2 h. Non‐target species (by‐catch) were released immediately. Twenty‐two sticklebacks were caught per site, excluding one site (PL3) where 11 fish were caught (n_total_ = 99). Fish were immediately transferred to individual plastic containers with 700 mL of filtered (0.2 μm) water from the site. These containers were set in a shallow area of the site to maintain their temperature. The fish were incubated for 120 min to measure their excretion. 30 mL water samples were taken using a syringe at times 0, 40, 80 and 120 min after mixing the water to ensure homogenisation (Durston and El‐Sabaawi 2019; Whiles et al. 2009). After excretion measurements, fish were individually euthanised via clove oil (500 mg/L) immersion followed by cervical dislocation. The euthanised sticklebacks were put on ice in the field and immediately frozen at −20°C upon returning to the laboratory. Environmental data including temperature, pH, turbidity, dissolved oxygen (DO), and conductivity were also collected at all sites (Table S1). Three water samples were collected from each site near where the traps were set in the littoral zone, filtered through a 25 mm glass filter and subsequently frozen for further laboratory analysis of soluble reactive phosphorus (SRP) and ammonium (Table S1).

### 2.3 Morphological Measurements

Fish were thawed in lukewarm water for 20 min. Morphological measurements, including standard length, were taken using electronic callipers within an accuracy of 0.01 mm (Tables S2–S4). The lateral plates were counted, and three armour phenotypes were assigned: completely plated (29–32 plates), partially plated (8–28 plates, in the anterior and keel regions), or low plated (5–7 armour plates, only in the anterior region) (Wiig et al. 2016).

Condition index (CI) was calculated for all fish by using the ratio of the measured dry weight to the weight predicted by its length (Garrow and Webster 1985). The condition index is used as a proxy for ‘fattiness’ or lipid content. However, condition may also reflect factors other than lipid stores, such as protein and muscle content (Wilder et al. 2016). Fish with a CI greater than 100% have a heavier weight per unit length than expected based on length, and so are considered relatively fattier (i.e., in ‘better’ condition); the opposite is true for fish with CI lower than 100% (Garrow and Webster 1985). Fish with a CI of 100% are considered normal, as their weight is what is expected given their length.

### 2.4 Body and Gut Stoichiometry

Stomach (foregut) contents were used as a proxy for diet quality, while hindgut (last 10 mm of the gut) contents were used as a proxy for the quality of egesta (May and El‐Sabaawi 2022a, 2022b; Sterner and George 2000). Not all fish had foregut and hindgut contents, so our sample sizes for these metrics are lower than the total fish sampled (Table S3). The P digestion ratio was calculated by taking the natural logarithm of the %P hindgut / %P foregut. This ratio provides information on P absorption in the gut by comparing the proportion of P in the foregut to the hindgut. Based on previously collected data, it was expected that sticklebacks would have relatively higher %P in the hindguts than foreguts (absorption ratio of > 0) perhaps because they absorb C and N more readily than P (May and El‐Sabaawi 2022a, 2022b). Fish investing more heavily in bone (fully plated morphs) might have a relatively more negative (i.e., smaller) digestion ratio (compared to low plated fish) if they are preferentially assimilating dietary P.

After drying at 50°C for at least 1 week, the body of each fish (excluding the pelvic girdle, 5th and 7th lateral plates, gut contents, reproductive tissues, and viscera) was weighed (dry weight) and ground into powder using Retsch Mixer Mill MM 400. The pelvic girdle and 5th and 7th lateral plates were retained for future studies on bone mineral content. Most females were gravid, and eggs were retained for future studies on reproductive investment. The foregut and hindgut samples were ground by hand in Eppendorf tubes using tweezers.

The body, foregut, and hindgut powdered samples were analysed for %P using the acid molybdate method (Boros and Mozsár 2015; Durston and El‐Sabaawi 2019). All powder samples were run alongside a set of spinach (NIST1570a) and bonemeal (NIST1485) internal standards. The %P values were calculated using the dry tissue mass and corrected for extraction efficiency (which was > 95%). A subset of the body powder samples was analysed for %N at UBC's stable isotope facility. The N:P of the body was expressed as molar ratios and transformed using the natural logarithm to avoid ratio bias (Isles 2020).

### 2.5 Excretion Stoichiometry

Ammonium concentration in the excreta was measured using the fluorometric method (Taylor et al. 2007). Because ammonium is unstable during freeze–thaw cycles and when exposed to light, measurements were taken in dim conditions, following the first defrost only (Avanzino and Kennedy 1993; Taylor et al. 2007). Phosphorus excretion rate was determined spectrophotometrically, using the acid molybdate method to measure SRP concentration (Boros and Mozsár 2015; Durston and El‐Sabaawi 2019). Excretion rates, expressed in μg/min of elemental P and N, were determined using the concentration at 0 min as a baseline nutrient concentration (Durston and El‐Sabaawi 2019; Whiles et al. 2009). The N:P of excretion was expressed in the molar ratio and transformed using the natural logarithm to avoid ratio bias (Isles 2020).

### 2.6 Statistical Analysis

All data were analysed and visualised using R (R Core Team 2021). Shapiro–Wilk normality tests, normal qq‐plots, and residual plots were used to determine if the residuals were normally distributed and showed equal variance. The normally distributed variables (body %P, ln(P excretion rate), and ln(N:P excretion rate)) were assessed using general linear mixed effects models (GLMMs) constructed using the *LMER* package (Bates et al. 2015) (Table S5). We constructed global model predictors based on our hypotheses (Table S5). Because sticklebacks are sexually dimorphic in terms of body size and shape, and potentially diet (Aguirre et al. 2008; Reimchen et al. 2016), sex was included as a fixed effect in all GLMMs (Table S5). Our predictors for body %P and N:P included sex, plate morph, and condition index (Tables S5–S7). The predictors for the gut metrics included sex, plate morph, and gut length (Table S5). Phosphorus excretion rate was initially non‐normally distributed, so we normalised the residuals by transforming the data using the natural logarithm. The predictors for the natural log transformed P excretion rate were sex, standard length, and body %P (Tables S5 and S8), and the predictors for natural log transformed N:P excretion rate included sex, standard length, and body N:P (Tables S5 and S9). Two outliers were removed from the excretion dataset because they showed overall negative P excretion rates, which is likely not physiologically possible. These values suggest that the analysis was not effective (e.g., because of potential contamination of the baseline sample). All models were assessed for heteroscedasticity using a Levene's test; none showed evidence of unequal variance (*p* > 0.1).

Site was included as a random effect in the excretion GLMMs (Table S5). Site encompasses many environmental variables, such as temperature, salinity, water chemistry, and predation pressure. While we did measure some of these variables directly, none had clear relationships or explained the variation in body %P or excretion rate in exploratory analyses (not shown). Site as a category encompassed more environmental variation than any single environmental variable. All global models were checked for collinearity via variance inflation factor (VIF) scores, which were < 3 (Bartoń 2020). The top GLMMs were chosen using model selection by deploying the ‘dredge’ function from the *MuMIn* package on the global models (Bartoń 2020). The resulting models were ranked based on the Akaike information criterion corrected for small sample size (AICc). Models that were within ΔAICc < 2 of the top model were averaged using the *model.avg* function in the *MuMIn* package (Bartoń 2020).

The gut metrics (%P foregut, P digestion ratio and %P hindgut) were not normally distributed, and their distributions were not improved by transformation (natural logarithm, square root transformations, etc.). Therefore, differences among the armour phenotypes in gut metrics were analysed using Kruskal‐Wallis tests. While we hypothesised that foregut %P, P digestion ratio, and hindgut %P would affect P and N:P excretion rate, we did not include these in our initial global models because including them would considerably reduce the overall sample size and therefore our confidence in the results. However, we re‐ran model selection with and without the gut metrics to see if they affected our findings. We found that the model selection results were similar (Tables S10–S13).

## 3 Results

### 3.1 Morphological and Elemental Variation

Stickleback morphology varied substantially in the Sooke River sticklebacks. As predicted, stickleback lateral plate number varied along the Sooke River continuum. Fully plated fish inhabited the estuary (SRC) and lower river site (SRR), and low plated fish were found in the upper river sites (PL3 and URC) (Figure 2). The middle site (PL1) contained all three plate morphs in nearly even proportions (Figure 2). Other morphometric variables such as standard length, dry mass, gut length, pelvic girdle length, and condition index varied substantially in the Sooke River (Table S2). Standard length varied from 36.41 to 68.12 mm (Table S2). Generally, larger fish with greater gut lengths and longer pelvic girdles inhabited the estuary (analyses not shown). Condition index varied from 55.53% to 186.75%, and while this variation was substantial (CV = 22.63%), it was not clear that it varied along the continuum (Table S2).

### 3.2 Body %P and N:P

**FIGURE 3:**
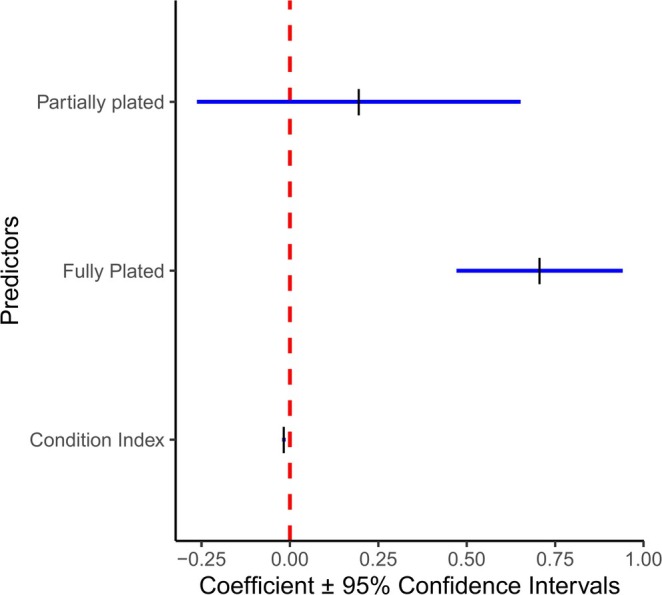
Regression coefficient plot for body %P. Vertical dashes indicate the coefficient for each predictor in the body %P model (Table 1). Positive values indicate positive effect of predictor on body %P, while negative values indicate a negative effect. The horizontal lines indicate 95% confidence intervals. The dashed line indicates the point at which a predictor has no effect.

**FIGURE 4:**
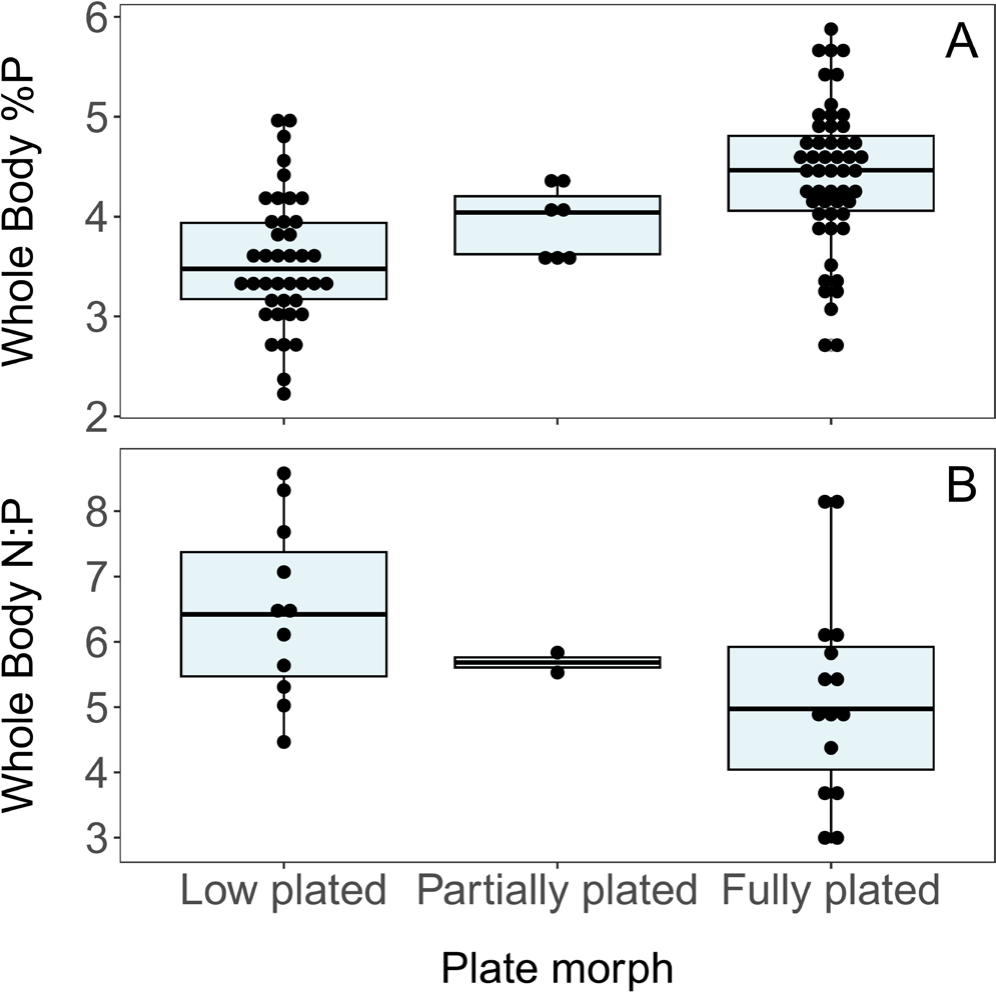
Box plot showing the relationship between lateral plate morphology and whole body %P (A) and whole body N:P (B) in the Sooke River sticklebacks. Boxes represent the interquartile range and horizontal lines are medians. Dots are binned to indicate number of samples within each value and represent individual fish.

Body %P ranged from 2.23% to 5.88% (Table S3). The model that best explained the variation in body %P included the fixed effects of plate morphology and condition index (Table 1). Lateral plate morphology had the largest effect on body %P (Figure 3). Fully plated fish had the highest body %P, low plated fish had the lowest, and partially plated fish had intermediate values (Figures 3 and 4A and Table 1). Condition had a statistically significant but small negative effect on body %P (Figure 3, Table 1 and Figure S1).

**TABLE 1:** Summary of the top model for body %P. Model predictors include plate morph, condition index. *R* ^2^ = 0.50, Adjusted *R* ^2^ = 0.49. The reference category was the low plated plate morph. The model was chosen based on a model selection procedure described in the methods.

	Estimate	SD	*p*
Intercept	6.07	0.26	< 0.001
Plate morph (partially plated)	0.20	0.23	0.41
Plate morph (fully plated)	0.71	0.12	< 0.001
Condition Index	−0.017	0.0026	< 0.001

Body %N ranged from 6.30% to 15.40%, and body N:P ranged from 2.91 to 8.58 (Table S3). Model selection on body N:P showed that the null model was the best model, suggesting that none of the predictors had an appreciable effect on body N:P (analyses not shown). However, there was a tendency for body N:P to vary with plate morph, with fully plated fish having lower N:P ratios than low‐plated fish (Figure 4B).

### 3.3 Gut Analyses

**FIGURE 5:**
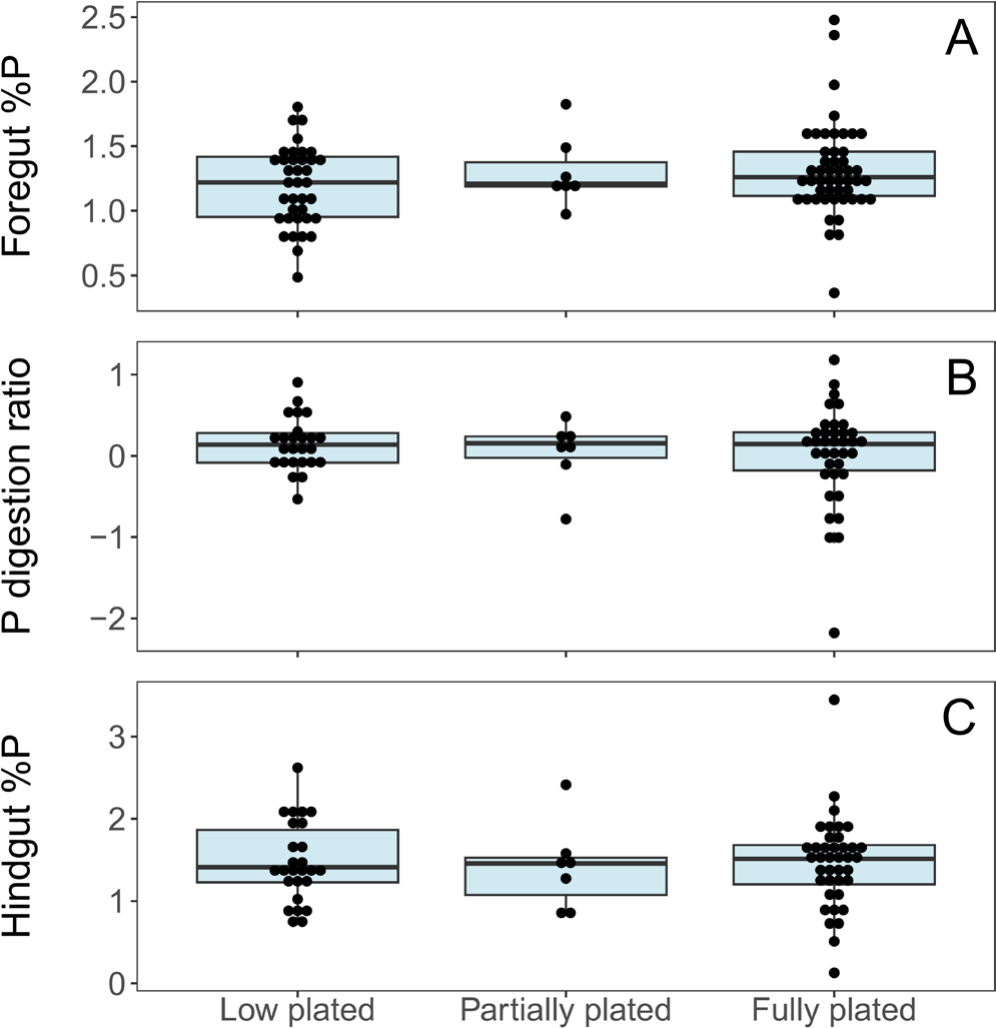
Box plot showing the relationship between lateral plate morphology and gut contents in the Sooke River sticklebacks. Boxes represent the interquartile range and horizonal lines are medians. Dots are binned to indicate number of samples within each value and represent individual fish. (A) The foregut %P, which proxies diet %P, (B) The digestion ratio, which proxies the relative absorption of P in the gut, (C) The hindgut %P, which proxies egestion %P.

Diet %P (proxied by foregut contents) ranged from 0.36% to 2.48% while egesta %P (proxied by hindgut contents) ranged from 0.13% to 3.45% (Table S3). As predicted, foregut %P was higher in fully plated fish than in low plated, but this difference was not statistically significant according to a Kruskal‐Wallis test (*p* = 0.41) (Figure 5A). Foregut %P was significantly higher in females than in males according to a Kruskal‐Wallis test (*p* = 0.019) (Figure S2). Contrary to our predictions, there were no trends found in either the digestion ratio (proxy for absorption) (Figure 5B), nor in hindgut %P (proxy for egestion) (Figure 5C).

### 3.4 Excretion

**FIGURE 6:**
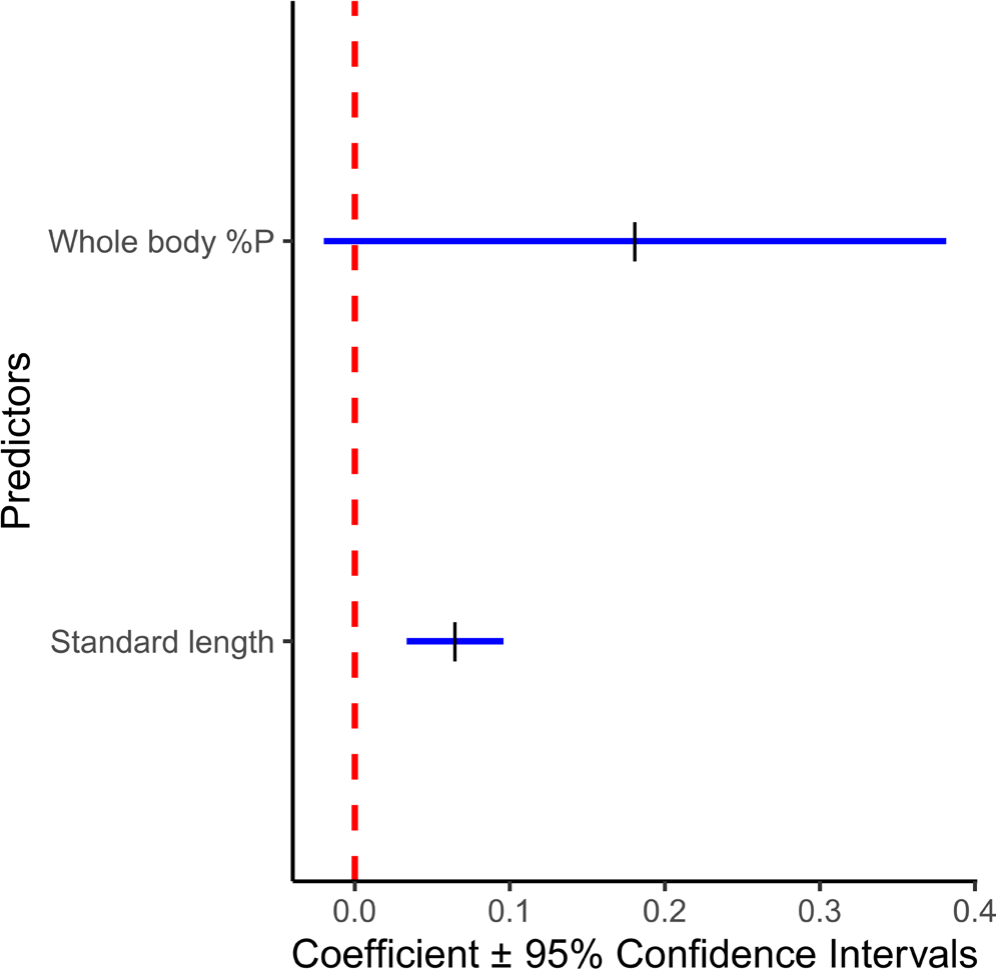
Regression coefficient plot for the averages of the top selected model for ln transformed P excretion rate. Dashes indicate the coefficient for each predictor in the P excretion rate model (Table 2). Positive values indicate positive effect of predictor on body %P, while negative values indicate a negative effect. The horizontal lines indicate 95% confidence intervals. The dashed line indicates the point at which a predictor has no effect.

Our three contrasting predictions concerning excretion were that (1) P excretion would have no relationship to body %P if increased P assimilation occurs; (2) P excretion rate would be negatively related to body %P if preferential feeding occurs (foregut %P will be positively related to body %P); or (3) P excretion would have a positive relationship with body %P in support of the VBH. We further predicted that P excretion rate would be positively related to standard length. We found that the model that best explained the variation in excretion P included standard length and body %P (Table 2). As predicted, standard length was positively related to P excretion rate. It had a relatively small effect, but its inclusion improved the fit of the model (Figure 6). Body %P was positively related to P excretion rate, which lends support to the VBH (Figure 6). However, its effects were relatively small, and its confidence intervals overlap with the zero‐effect line (Figure 6 and Table 2).

**TABLE 2:** Summary of averaged top model for P excretion rate (ug/min) and N:P excretion. Both were transformed using the natural log prior to analysis. Model predictors for P excretion rate include standard length, body %P, and site as a random effect (marginal *R* ^2^ = 0.15, conditional *R* ^2^ = 0.63). Model predictors for N:P of excretion are body N:P and site as a random effect (marginal *R* ^2^ = 0.0018, conditional *R* ^2^ = 0.84). See 2. Methods for description of model selection procedure.

Model	Predictors	Estimate	SD	*p*
	Intercept	−5.48	0.84	< 0.001
Ln(P excretion rate)	Standard length	0.065	0.016	< 0.001
	Body %P	0.18	0.10	0.077
Ln(N:P excretion rate)	Intercept	2.75	0.60	< 0.001
	Body N:P	0.037	0.18	0.84

The best model for N:P excretion was the null, suggesting that none of our predictors were important (Table S9). However, excretion N:P was very weakly positively correlated with body N:P (Table 2).

## 4 Discussion

Because freshwater fish may represent the largest pools of N and P in aquatic environments (Kitchell et al. 1975) and can have substantial impacts on nutrient cycles and community composition (Griffiths 2006; Kitchell et al. 1975; McIntyre et al. 2008; Vanni and Layne 1997), it is important to understand what drives variation in their OS, and how that variation relates to the elemental content of the diet and the waste. We explored controls on vertebrate‐driven nutrient recycling using sticklebacks as a model and ES as a framework. Here we show that sticklebacks within a single river watershed display substantial morphological and stoichiometric variation. Specifically, variation in adaptive bony armour (plate phenotype) traits drove variation in OS and excretion. Our study further suggests that although diet and egestion display considerable variation and interesting patterns, they do not necessarily have significant relationships with body %P or bony phenotype. Nonetheless, we found that P excretion was positively related to body %P, supporting predictions from the VBH (May and El‐Sabaawi 2024).

We found considerable variation in the Sooke River stickleback morphology (Tables S4 and S10), comparable to what is found in studies conducted across much larger geographical regions. Standard length ranged from 36.4 to 68.1 mm, which is greater than the range (36–56 mm) found by Durston and El‐Sabaawi (2017) in their survey of 375 sticklebacks from 12 populations from across BC. Body condition varied from 55.53%–186.75%, which resembles the range found (59.71%–203.8%) in a study that sampled threespine sticklebacks from 15 sites along the California coast (Appleby 2016—unpublished data). We found that lateral plate morphology varied along the Sooke River as we might expect based on the importance of the marine to freshwater transition for armour phenotype (Bell and Foster 1994). We also found that larger fish with longer pelvic girdles inhabited the estuary, a pattern that was also found in a study on stickleback populations along the River Tyne (Jones et al. 2006). We also identified a clear hybrid zone in the river where full, low, and partially plated fish were found (Figure 2). This hybrid zone has not been previously described; future research could further explore the stickleback morphology at this site. Hybrid zones can teach us how bone phenotype alters OS and excretion without the added variable of different sites. Regardless of what drives selection on lateral plate phenotype, stickleback evolution has led to site‐associated differences in bone investment, which may lead to different ecological consequences for each of these locations. These broad ranges in morphology and bone investment suggest that rivers are interesting systems within which to study intraspecific phenotypic variation in stickleback morphology.

Stickleback OS varied considerably (Table S3), and lateral plate morphology and body condition explained some of this variation (Figures 3 and 4 and Table 1). The range of stickleback body %P and N:P (%P: 2.2%–5.9%; N:P: 2.91–8.585) is comparable to the range (%P: 3.1–6.2; N:P: 3–9) found by Durston and El‐Sabaawi (2017) in a study of 10 freshwater stickleback populations across Southern BC. These comparisons show that the Sooke watershed sticklebacks have variation in OS comparable to what is observed over a much larger scale. Lateral plate morphology had a large effect on body %P (Figure 3), where fully plated fish had a higher %P and lower N:P than low plated fish (Figure 4). This supports the prediction that those investing more heavily in bony armour store proportionally more P. It is important to test the relationship between armour and %P in each system because they are not always correlated. Some studies have observed this correlation (Durston and El‐Sabaawi 2017; El‐Sabaawi et al. 2016), while others have not (Rudman et al. 2019). It is currently unknown why this relationship varies, but it suggests that sticklebacks may be using a variety of mechanisms to build their armour, including reallocating P from other structures (El‐Sabaawi et al. 2023; Leal et al. 2017). Our results also show that body condition can noticeably change the proportion of P in the body. Condition is negatively correlated with %P, likely because factors such as increased fat content increase condition. Fat is high in C, so would dilute the proportion of P in the fish body, leading to this negative relationship (Sterner and Elser 2002).

In a field study, it is challenging to measure diet quality, yet these metrics could potentially explain why much empirical data on vertebrates does not support ES predictions. Here we use proxies that have not been broadly used in other studies, making this approach interesting. The elemental composition of stickleback diets varied considerably within the Sooke River (Figure 5A). Diet %P (proxied by foregut contents) had a greater range (0.36%–2.48%) than that found (0.18%–1.82%) in a study that caught 94 wild sticklebacks from the Sooke estuary and Miami creek (May and El‐Sabaawi 2022a). Sticklebacks typically eat zooplankton and macroinvertebrates (Arnegard et al. 2014), which likely vary in %P from ~0.61% to 1.24% (Archambeault et al. 2020). Our gut content results suggests that stickleback diet is more varied than previously thought, and that sticklebacks are likely eating materials ranging from detritus to juvenile fish (Frost et al. 2006; McManamay et al. 2011). Future studies could identify prey in the gut and conduct DNA metabarcoding to further investigate the source of this broad range in diet quality (Buckland et al. 2017; Jakubavičiūtė et al. 2017).

Prey selection is very important, and is potentially a way that animals can meet their elemental demands (El‐Sabaawi et al. 2023). We proposed that fully plated fish might be able to meet their dietary P demand by selectively foraging on P‐rich prey. While we did not perform prey selection experiments, we found that diet %P was marginally higher in fully plated fish. There are several possible explanations for why we did not see a stronger relationship between diet %P and bone investment. Consumption rate is another important dietary metric that could mask altered excretion (Moody et al. 2018). Because consumption rate cannot be accurately measured in a field setting, we cannot discount the possibility that fully plated fish have higher consumption rates. Another possibility is that dietary P may not be limiting to the fish and therefore there would be no pressure to preferentially ingest or absorb P, which would explain the lack of a strong relationship between bone investment and dietary P (Archambeault et al. 2020). This would also support the hypothesis that dietary P is not limiting in freshwater fish (Boersma and Elser 2006; Hood et al. 2005; Schindler and Eby 1997; Vrede et al. 2004). Alternatively, it is possible that our sample size may not have been large enough to adequately show that fish investing more heavily in bone preferentially feed on higher P prey. Future studies could quantify prey availability to uncover if sticklebacks may be preferentially feeding on certain items.

Seasonality is another important consideration when examining fish diets. Diet %P was significantly higher in females than in males, indicating that perhaps females have a higher quality diet. Previous work on sticklebacks has found significant differences in the isotopic C and N signatures of males and females, suggesting differences in foraging (Reimchen et al. 2016). During the breeding season, males must guard nests and remain in a more littoral niche, which may influence foraging behaviour and diet, potentially causing these differences in diet (Reimchen et al. 2016). Furthermore, during the breeding season, stickleback egg cannibalism is common (Allen and Wootton 1984; Wootton 1976), and we observed eggs in fish guts during dissections. If a large portion of the summer diet is eggs, then it may not be representative of typical foraging habits, which may or may not include selective foraging. Differences in diet quality between the plate morphs and the sexes merit further study and could be examined during different seasons to identify if reproduction induces dietary shifts.

Variation in assimilation into the tissues and absorption in the gut can be very important, as these metrics are difficult to measure, yet could mask altered excretion. The P digestion ratio and the egestion %P (proxied by hindgut %P) varied substantially but were not correlated with plate morphology (Figure 3B,C), gut length, or OS. Egestion %P ranged from 0.13 to 3.45 %P—a broader range than diet %P. This suggests that there is considerable variation in how nutrients are absorbed in the gut. We found that the P digestion ratio, which proxies nutrient absorption in the gut, ranged from −2.18 to 1.18, which is similar to the digestion ratios found by May and El‐Sabaawi (2022a, 2022b). We found that 65% of the fish had a positive digestion ratio, indicating that many fish contain relatively higher %P in their hindguts than foreguts. This could mean that other elements from the diet such as C or N are preferentially absorbed in the gut, leading to a proportionally higher relative concentration of P in the hindgut (May and El‐Sabaawi 2024—unpublished results). This may be the case, as C is needed in relatively larger quantities for metabolism and reproduction (Frost et al. 2006). Alternatively, dietary P sources such as fish bones or plant material may not be as easily digested as dietary sources of C and N. Interestingly, fully plated fish had higher variation in diet %P, P digestion ratio, and egestion %P than partially and low plated fish. This may indicate that fully plated fish have a more varied diet and have more variation in how they absorb nutrients in their guts and should be further explored.

Phosphorus excretion rates were positively related to OS and standard length (Figure 6 and Table 2). Furthermore, the N:P of excretion was positively related to body N:P (Table 2). Body size generally predicts many metabolic rates (Peters 1983), and a synthesis that reviewed over 10,000 observations of aquatic animal P excretion suggested that body size is a principal predictor of N and P excretion rates (Vanni and McIntyre 2016). Our data further support these findings. Our data also suggest that fish investing more in bone have a higher body %P, and that those fish excrete P at a higher rate. This implies that individuals with relatively *more* bone may be *less* limited by P. A similar relationship was observed in threespine sticklebacks reared under laboratory conditions in a common garden experiment, where fully plated fish excreted significantly higher P than low plated fish (Rudman et al. 2019). Bone is a stoichiometrically flexible tissue that can store and mobilise different minerals to maintain mineral homeostasis (Pasteris et al. 2008). Therefore, the essential ES assumption of body elemental homeostasis may be broken in vertebrates (May and El‐Sabaawi 2024). Indeed, others have noted that there is variation in the degree to which fish maintain N and P homeostasis (Benstead et al. 2014; Persson et al. 2010). If bone can store and release P, then it is possible that the relatively greater proportion of bone in fully plated fish acts as a store of P that buffers against dietary limitation and lowers their overall P demand (May and El‐Sabaawi 2024). It is therefore important for future studies to assess the role of bone in homeostasis and how this relates to P demand and release.

Alternative explanations for the trends in excretion rates are possible, but methodological improvements are required to investigate these possibilities. To properly make predictions about waste stoichiometry, we require information about diet quality, physiology, body size, and OS. However, in a field study, most studies can only reliably measure OS and body size. In this paper, we used proxies for diet, absorption, and egesta, and these methods might be too coarse to properly capture potentially stronger trends. For example, P assimilation efficiency has been shown to influence excretion in theoretical studies (Schindler and Eby 1997), but assimilation cannot be calculated using our proxies. Assimilation efficiency in sticklebacks has been measured in the lab using radioisotopes (Rudman et al. 2019), but these methods are difficult to use in a field setting and require both full gut clearance and accurate dietary information (May and El‐Sabaawi 2022a, 2022b). Therefore, to test ES predictions properly in natural ecosystems, we must continue to develop and test methods that can capture physiological information in a field setting.

Our study highlights the wealth of information that can be gained by studying intraspecific variation in stoichiometry and excretion within a single species in a river ecosystem.

The observed relationship between body P content and P excretion rate provides a foundation for future research into the mechanisms governing vertebrate excretion. This information could prove invaluable as we move towards making predictions in how vertebrate evolution, variation, as well as abundance and diversity declines can influence nutrient recycling in nature.

## Author Contributions

**Sarah R. Rozanski:** conceptualization (lead), data curation (equal), formal analysis (equal), funding acquisition (lead), investigation (supporting), methodology (lead), project administration (supporting), visualization (supporting), writing – original draft (lead), writing – review and editing (equal). **Emily M. May:** conceptualization (supporting), data curation (equal), formal analysis (equal), methodology (equal), project administration (supporting), validation (equal), visualization (supporting), writing – review and editing (equal). **Rana W. El‐Sabaawi:** conceptualization (lead), data curation (equal), formal analysis (equal), funding acquisition (lead), investigation (lead), methodology (equal), project administration (lead), resources (lead), supervision (lead), validation (equal), visualization (equal), writing – original draft (supporting), writing – review and editing (equal).

## Ethics Statement

Our research was performed in accordance with the Canadian Council for Animal Care guidelines. Sticklebacks were sampled in accordance with the Ministry of Environment (NA23‐788740) and Fisheries and Oceans Canada (XR 1162023) permits. Fish handling and euthanasia were completed in accordance with an Animal Use Protocol approved by UVic's Animal Care Committee (2022‐005(2)).

## Conflicts of Interest

The authors declare no conflicts of interest.

## Supporting information


**Figure S1:** Scatter plot showing the relationship between body %P and condition index. Dots represent data collected from an individual fish and shaded areas are 95% confidence intervals. Body %P declines with increasing body condition in the Sooke River sticklebacks.

**Figure S2:** Box plot showing the differences in foregut %P between the sexes. Boxes represent the interquartile range and horizonal lines are medians. Dots are binned and represent individual fish.

**Data S1:** Summary of site, water quality, and environmental nutrient data.

## Data Availability

All data and code associated with this research is available on dryad (DOI: 10.5061/dryad.j6q573nqg) and will be published pending acceptance.

## References

[ece371920-bib-0001] Aguirre, W. E. , and M. A. Bell . 2012. “Twenty Years of Body Shape Evolution in a Threespine Stickleback Population Adapting to a Lake Environment: Stickleback Body Shape Evolution.” Biological Journal of the Linnean Society 105: 817–831. 10.1111/j.1095-8312.2011.01825.x.

[ece371920-bib-0002] Aguirre, W. E. , K. E. Ellis , M. Kusenda , and M. A. Bell . 2008. “Phenotypic Variation and Sexual Dimorphism in Anadromous Threespine Stickleback: Implications for Postglacial Adaptive Radiation: Variation and Sexual Dimorphism in Anadromous Stickleback.” Biological Journal of the Linnean Society 95: 465–478. 10.1111/j.1095-8312.2008.01075.x.

[ece371920-bib-0003] Allen, J. R. M. , and R. J. Wootton . 1984. “Temporal Patterns in Diet and Rate of Food Consumption of the Three‐Spined Stickleback (*Gasterosteus aculeatus* L.) in Llyn Frongoch, an Upland Welsh Lake.” Freshwater Biology 14: 335–346. 10.1111/j.1365-2427.1984.tb00158.x.

[ece371920-bib-0004] Allgeier, J. E. , C. A. Layman , P. J. Mumby , and A. D. Rosemond . 2014. “Consistent Nutrient Storage and Supply Mediated by Diverse Fish Communities in Coral Reef Ecosystems.” Global Change Biology 20: 2459–2472. 10.1111/gcb.12566. 24692262

[ece371920-bib-0005] Appleby, M. G. 2016. “Intraspecific Variation in Body Stoichiometry of Threespine Stickleback (Gasterosteus Aculeatus) in Bar‐Back Estuaries Along the Central Californian Coast.” University of Victoria.

[ece371920-bib-0006] Archambeault, S. L. , D. J. Durston , A. Wan , R. W. El‐Sabaawi , B. Matthews , and C. L. Peichel . 2020. “Phosphorus Limitation Does Not Drive Loss of Bony Lateral Plates in Freshwater Stickleback ( *Gasterosteus aculeatus* ).” Evolution 74: 2088–2104. 10.1111/evo.14044. 32537747 PMC7773418

[ece371920-bib-0007] Arnegard, M. E. , M. D. McGee , B. Matthews , et al. 2014. “Genetics of Ecological Divergence During Speciation.” Nature 511: 307–311. 10.1038/nature13301. 24909991 PMC4149549

[ece371920-bib-0008] Atkinson, C. L. , K. A. Capps , A. T. Rugenski , and M. J. Vanni . 2016. “Consumer‐Driven Nutrient Dynamics in Freshwater Ecosystems: From Individuals to Ecosystems: Consumer‐Driven Nutrient Dynamics in Freshwater Ecosystems.” Biological Reviews 92: 2003–2023. 10.1111/brv.12318. 28008706

[ece371920-bib-0009] Avanzino, R. J. , and V. C. Kennedy . 1993. “Long‐Term Frozen Storage of Stream Water Samples for Dissolved Orthophosphate, Nitrate Plus Nitrite, and Ammonia Analysis.” Water Resources Research 29: 3357–3362. 10.1029/93WR01684.

[ece371920-bib-0010] Barlak, R. 2019. “Water Quality Assessment and Proposed Objectives for Sooke Watersheds, Inlet, Harbour and Basin.”

[ece371920-bib-0011] Bartoń, K. 2020. “MuMIn: Multi‐Model Inference.”

[ece371920-bib-0012] Bassar, R. D. , M. C. Marshall , A. López‐Sepulcre , et al. 2010. “Local Adaptation in Trinidadian Guppies Alters Ecosystem Processes.” Proceedings of the National Academy of Sciences 107: 3616–3621. 10.1073/pnas.0908023107. PMC2840427 20133670

[ece371920-bib-0013] Bates, D. , M. Mächler , B. Bolker , and S. Walker . 2015. “Fitting Linear Mixed‐Effects Models Using lme4.” Journal of Statistical Software 67: i01. 10.18637/jss.v067.i01.

[ece371920-bib-0014] Bell, M. A. , and S. A. Foster . 1994. “The Evolutionary Biology of the Threespine Stickleback.” Journal of Animal Ecology 64: 418. 10.2307/5902.

[ece371920-bib-0015] Benstead, J. P. , J. M. Hood , N. V. Whelan , et al. 2014. “Coupling of Dietary Phosphorus and Growth Across Diverse Fish Taxa: A Meta‐Analysis of Experimental Aquaculture Studies.” Ecology 95: 2768–2777. 10.1890/13-1859.1.

[ece371920-bib-0016] Boersma, M. , and J. J. Elser . 2006. “Too Much of a Good Thing: On Stoichiometrically Balanced Diets and Maximal Growth.” Ecology 87: 1325–1330. 16761610 10.1890/0012-9658(2006)87[1325:tmoagt]2.0.co;2

[ece371920-bib-0017] Boros, G. , and A. Mozsár . 2015. “Comparison of Different Methods Used for Phosphorus Determination in Aquatic Organisms.” Hydrobiologia 758: 235–242. 10.1007/s10750-015-2293-2.

[ece371920-bib-0018] Brabrand, Å. , B. A. Faafeng , and J. P. Moritz Nilssen . 1990. “Relative Importance of Phosphorus Supply to Phytoplankton Production: Fish Excretion Versus External Loading.” Canadian Journal of Fisheries and Aquatic Sciences 47: 364–372. 10.1139/f90-038.

[ece371920-bib-0019] Buckland, A. , R. Baker , N. Loneragan , and M. Sheaves . 2017. “Standardising Fish Stomach Content Analysis: The Importance of Prey Condition.” Fisheries Research 196: 126–140. 10.1016/j.fishres.2017.08.003.

[ece371920-bib-0020] Burke, J. R. 2002. “Methods for Continuous Automated Turbidity Monitoring in British Columbia, Canada.”

[ece371920-bib-0021] Colosimo, P. F. , K. E. Hosemann , S. Balabhadra , et al. 2005. “Widespread Parallel Evolution in Sticklebacks by Repeated Fixation of Ectodysplasin Alleles.” Science 307: 1928–1933. 10.1126/science.1107239. 15790847

[ece371920-bib-0022] CRD . 2017. “Captial Regional District Integrated Water Services Website.”

[ece371920-bib-0023] Doughty, C. E. , J. Roman , S. Faurby , et al. 2016. “Global Nutrient Transport in a World of Giants.” Proceedings of the National Academy of Sciences of the United States of America 113: 868–873. 10.1073/pnas.1502549112. 26504209 PMC4743783

[ece371920-bib-0024] Durston, D. 2016. “How the Evolution of Bony Traits Influences Resource Interactions in Threespine Stickleback (Thesis).”

[ece371920-bib-0025] Durston, D. J. , and R. W. El‐Sabaawi . 2017. “Bony Traits and Genetics Drive Intraspecific Variation in Vertebrate Elemental Composition.” Functional Ecology 31: 2128–2137. 10.1111/1365-2435.12919.

[ece371920-bib-0026] Durston, D. J. , and R. W. El‐Sabaawi . 2019. “The Utility of Stoichiometric and Metabolic Theory for Understanding the Foraging Habitat and Excretion of Threespine Stickleback (*Gasterosteus aculeatus*).” Evolutionary Ecology Research 20: 193–211.

[ece371920-bib-0027] El‐Sabaawi, R. W. , K. D. Lemmen , P. D. Jeyasingh , and S. A. J. Declerck . 2023. “SEED: A Framework for Integrating Ecological Stoichiometry and Eco‐Evolutionary Dynamics.” Ecology Letters 26: S109–S126. 10.1111/ele.14285. 37840025

[ece371920-bib-0028] El‐Sabaawi, R. W. , M. C. Marshall , R. D. Bassar , A. López‐Sepulcre , E. P. Palkovacs , and C. Dalton . 2015. “Assessing the Effects of Guppy Life History Evolution on Nutrient Recycling: From Experiments to the Field.” Freshwater Biology 60: 590–601. 10.1111/fwb.12507.

[ece371920-bib-0029] El‐Sabaawi, R. W. , M. L. Warbanski , S. M. Rudman , R. Hovel , and B. Matthews . 2016. “Investment in Boney Defensive Traits Alters Organismal Stoichiometry and Excretion in Fish.” Oecologia 181: 1209–1220. 10.1007/s00442-016-3599-0. 27075487

[ece371920-bib-0030] Elser, J. J. , and J. Urabe . 1999. “The Stoichiometry of Consumer‐Driven Nutrient Recycling: Theory, Observations, and Consequences.” Ecology 80: 735–751.

[ece371920-bib-0031] Frost, P. C. , J. P. Benstead , W. F. Cross , et al. 2006. “Threshold Elemental Ratios of Carbon and Phosphorus in Aquatic Consumers.” Ecology Letters 9: 774–779. 10.1111/j.1461-0248.2006.00919.x. 16796566

[ece371920-bib-0032] Garrow, J. S. , and J. Webster . 1985. “Quetelet's Index (W/H2) as a Measure of Fatness.” International Journal of Obesity 9: 147–153. 4030199

[ece371920-bib-0033] Gillooly, J. F. , A. P. Allen , J. H. Brown , et al. 2005. “The Metabolic Basis of Whole‐Organism RNA and Phosphorus Content.” Proceedings of the National Academy of Sciences 102: 11923–11927. 10.1073/pnas.0504756102. PMC1187991 16091465

[ece371920-bib-0034] Griffiths, D. 2006. “The Direct Contribution of Fish to Lake Phosphorus Cycles.” Ecology of Freshwater Fish 15: 86–95. 10.1111/j.1600-0633.2006.00125.x.

[ece371920-bib-0035] Hagen, D. W. , and L. G. Gilbertson . 1972. “Geographic Variation and Environmental Selection in *Gasterosteus aculeatus* L. in the Pacific Northwest, America.” Evolution 26: 32–51. 10.2307/2406981. 28555771

[ece371920-bib-0036] Hall, S. R. 2009. “Stoichiometrically Explicit Food Webs: Feedbacks Between Resource Supply, Elemental Constraints, and Species Diversity.” Annual Review of Ecology, Evolution, and Systematics 40: 503–528. 10.1146/annurev.ecolsys.39.110707.173518.

[ece371920-bib-0037] Harmon, L. J. , B. Matthews , S. Des Roches , J. M. Chase , J. B. Shurin , and D. Schluter . 2009. “Evolutionary Diversification in Stickleback Affects Ecosystem Functioning.” Nature 458: 1167–1170. 10.1038/nature07974. 19339968

[ece371920-bib-0038] Hendrixson, H. A. , R. W. Sterner , and A. D. Kay . 2007. “Elemental Stoichiometry of Freshwater Fishes in Relation to Phylogeny, Allometry and Ecology.” Journal of Fish Biology 70: 121–140. 10.1111/j.1095-8649.2006.01280.x.

[ece371920-bib-0039] Hood, J. M. , M. J. Vanni , and A. S. Flecker . 2005. “Nutrient Recycling by Two Phosphorus‐Rich Grazing Catfish: The Potential for Phosphorus‐Limitation of Fish Growth.” Oecologia 146: 247–257. 16133197 10.1007/s00442-005-0202-5

[ece371920-bib-0040] Isles, P. D. F. 2020. “The Misuse of Ratios in Ecological Stoichiometry.” Ecology 101: e03153. 10.1002/ecy.3153. 32731303

[ece371920-bib-0041] Jakubavičiūtė, E. , U. Bergström , J. S. Eklöf , Q. Haenel , and S. J. Bourlat . 2017. “DNA Metabarcoding Reveals Diverse Diet of the Three‐Spined Stickleback in a Coastal Ecosystem.” PLoS One 12: e0186929. 10.1371/journal.pone.0186929. 29059215 PMC5653352

[ece371920-bib-0042] Jones, F. C. , C. Brown , J. M. Pemberton , and V. A. Braithwaite . 2006. “Reproductive Isolation in a Threespine Stickleback Hybrid Zone.” Journal of Evolutionary Biology 19: 1531–1544. 10.1111/j.1420-9101.2006.01122.x. 16910983

[ece371920-bib-0043] Karasov, W. H. , and A. E. Douglas . 2013. “Comparative Digestive Physiology.” In Comprehensive Physiology, edited by Y. S. Prakash , 741–783. Wiley. 10.1002/cphy.c110054. PMC4458075 23720328

[ece371920-bib-0044] Kitchell, J. F. , J. F. Koonce , and P. S. Tennis . 1975. “Phosphorus Flux Through Fishes: With 2 Figures and 3 Tables in Text.” SIL Proceedings, 1922–2010 19: 2478–2484. 10.1080/03680770.1974.11896332.

[ece371920-bib-0045] Leal, M. C. , R. J. Best , D. Durston , R. W. El‐Sabaawi , and B. Matthews . 2017. “Stoichiometric Traits of Stickleback: Effects of Genetic Background, Rearing Environment, and Ontogeny.” Ecology and Evolution 7: 2617–2625. 10.1002/ece3.2802. 28428852 PMC5395448

[ece371920-bib-0046] Lemmen, K. D. , O. M. Butler , T. Koffel , S. M. Rudman , and C. C. Symons . 2019. “Stoichiometric Traits Vary Widely Within Species: A Meta‐Analysis of Common Garden Experiments.” Frontiers in Ecology and Evolution 7: 339. 10.3389/fevo.2019.00339.

[ece371920-bib-0047] May, E. , and R. El‐Sabaawi . 2022a. “Measuring Egestion, Excretion, Fecal Leaching, and Foregut‐Hindgut %P in Threespine Stickleback.”

[ece371920-bib-0048] May, E. M. , and R. W. El‐Sabaawi . 2022b. “Comparing Methods for Measuring Egestion in Aquatic Animals.” Limnology and Oceanography: Methods 20: 721–728. 10.1002/lom3.10516.

[ece371920-bib-0049] May, E. M. , and R. W. El‐Sabaawi . 2024. “The Vertebrate Bone Hypothesis: Understanding the Impact of Bone on Vertebrate Stoichiometry.” Functional Ecology 38: 14509. 10.1111/1365-2435.14509.

[ece371920-bib-0050] McIntyre, P. B. , and A. S. Flecker . 2010. “Ecological Stoichiometry as an Integrative Framework in Stream Fish Ecology.”

[ece371920-bib-0051] McIntyre, P. B. , A. S. Flecker , M. J. Vanni , J. M. Hood , B. W. Taylor , and S. A. Thomas . 2008. “Fish Distributions and Nutrient Cycling in Streams: Can Fish Create Biogeochemical Hotspots.” Ecology 89: 2335–2346. 10.1890/07-1552.1. 18724743

[ece371920-bib-0052] McIntyre, P. B. , L. E. Jones , A. S. Flecker , and M. J. Vanni . 2007. “Fish Extinctions Alter Nutrient Recycling in Tropical Freshwaters.” Proceedings of the National Academy of Sciences 104: 4461–4466. 10.1073/pnas.0608148104. PMC1838623 17360546

[ece371920-bib-0053] McManamay, R. A. , J. R. Webster , H. M. Valett , and C. A. Dolloff . 2011. “Does Diet Influence Consumer Nutrient Cycling? Macroinvertebrate and Fish Excretion in Streams.” Journal of the North American Benthological Society 30: 84–102. 10.1899/09-152.1.

[ece371920-bib-0054] Moody, E. K. , E. W. Carson , J. R. Corman , et al. 2018. “Consumption Explains Intraspecific Variation in Nutrient Recycling Stoichiometry in a Desert Fish.” Ecology 99: 1552–1561. 10.1002/ecy.2372. 29882955

[ece371920-bib-0055] Pasteris, J. D. , B. Wopenka , and E. Valsami‐Jones . 2008. “Bone and Tooth Mineralization: Why Apatite?” Elements 4: 97–104. 10.2113/GSELEMENTS.4.2.97.

[ece371920-bib-0056] Pendray, T. 1980. “Sooke River Aquatics Inventory.” British Columbia Ministry Environment Assessment and Planning Division.

[ece371920-bib-0057] Persson, J. , P. Fink , A. Goto , J. M. Hood , J. Jonas , and S. Kato . 2010. “To Be or Not to Be What You Eat: Regulation of Stoichiometric Homeostasis Among Autotrophs and Heterotrophs.” Oikos 119: 741–751. 10.1111/j.1600-0706.2009.18545.x.

[ece371920-bib-0058] Peters, R. H. 1983. The Ecological Implications of Body Size, Cambridge Studies in Ecology. Cambridge University Press.

[ece371920-bib-0059] Reimchen, T. E. , D. Steeves , and C. A. Bergstrom . 2016. “Sex Matters for Defence and Trophic Traits of Threespine Stickleback.” Evolutionary Ecology Research 17: 459–485.

[ece371920-bib-0060] Rudman, S. M. , J. M. Goos , J. B. Burant , et al. 2019. “Ionome and Elemental Transport Kinetics Shaped by Parallel Evolution in Threespine Stickleback.” Ecology Letters 22: 645–653. 10.1111/ele.13225. 30724019

[ece371920-bib-0061] Rudman, S. M. , M. A. Rodriguez‐Cabal , A. Stier , et al. 2015. “Adaptive Genetic Variation Mediates Bottom‐Up and Top‐Down Control in an Aquatic Ecosystem.” Proceedings of the Royal Society B: Biological Sciences 282: 20151234. 10.1098/rspb.2015.1234. PMC4528534 26203004

[ece371920-bib-0062] Schindler, D. E. , and L. A. Eby . 1997. “Stoichiometry of Fishes and Their Prey: Implications for Nutrient Recycling.” Ecology 78: 1816–1831.

[ece371920-bib-0063] Schluter, D. 1993. “Adaptive Radiation in Sticklebacks: Size, Shape, and Habitat Use Efficiency.” Ecology 74: 699–709. 10.2307/1940797.

[ece371920-bib-0064] Schluter, D. 2000. The Ecology of Adaptive Radiation. OUP Oxford.

[ece371920-bib-0065] Sereda, J. M. , and J. J. Hudson . 2011. “Empirical Models for Predicting the Excretion of Nutrients (N and P) by Aquatic Metazoans: Taxonomic Differences in Rates and Element Ratios: Predicting Release of Nutrients by Aquatic Metazoans.” Freshwater Biology 56: 250–263. 10.1111/j.1365-2427.2010.02491.x.

[ece371920-bib-0066] Sereda, J. M. , J. J. Hudson , and P. D. Mcloughlin . 2008. “General Empirical Models for Predicting the Release of Nutrients by Fish, With a Comparison Between Detritivores and Non‐Detritivores.” Freshwater Biology 1: 497. 10.1111/j.1365-2427.2008.02029.x.

[ece371920-bib-0067] Sterner, R. W. , and J. J. Elser . 2002. Ecological Stoichiometry: The Biology of Elements From Molecules to the Biosphere. Princeton University Press.

[ece371920-bib-0068] Sterner, R. W. , and N. B. George . 2000. “Carbon, Nitrogen, and Phosphorus Stoichiometry of Cyprinid Fishes.” Ecology 81: 127–140. 10.1890/0012-9658(2000)081[0127:CNAPSO]2.0.CO;2.

[ece371920-bib-0069] Sterrett, S. C. , J. C. Maerz , and R. A. Katz . 2015. “What Can Turtles Teach Us About the Theory of Ecological Stoichiometry?” Freshwater Biology 60: 443–455. 10.1111/fwb.12516.

[ece371920-bib-0070] Taylor, B. W. , C. F. Keep , R. O. Hall , et al. 2007. “Improving the Fluorometric Ammonium Method: Matrix Effects, Background Fluorescence, and Standard Additions.” Journal of the North American Benthological Society 26: 167–177.

[ece371920-bib-0071] Thompson, C. E. , E. B. Taylor , and J. D. McPhail . 1997. “Parallel Evolution of Lake‐Stream Pairs of Threespine Sticklebacks (Gasterosteus) Inferred From Mitochondrial DNA Variation.” Evolution 51: 1955. 10.2307/2411016. 28565100

[ece371920-bib-0072] T'Sou‐ke Nation . 2021. “Welcome to T'sou‐Ke Nation.”

[ece371920-bib-0073] Vanni, M. J. , and C. D. Layne . 1997. “Nutrient Recycling and Herbivory as Mechanisms in the ‘Top–Down’ Effect of Fish on Algae in Lakes.” Ecology 78: 21–40.

[ece371920-bib-0074] Vanni, M. J. , and P. B. McIntyre . 2016. “Predicting Nutrient Excretion of Aquatic Animals With Metabolic Ecology and Ecological Stoichiometry: A Global Synthesis.” Ecology 97: 3460–3471. 10.1002/ecy.1582. 27912023

[ece371920-bib-0075] Vrede, T. , D. R. Dobberfuhl , S. A. L. M. Kooijman , and J. J. Elser . 2004. “Fundamental Connections Among Organism C:N:P Stoichiometry, Macromolecular Composition, and Growth.” Ecology 85: 1217–1229. 10.1890/02-0249.

[ece371920-bib-0076] Wei, H. , Y. Liang , Q. Luo , D. Gu , X. Mu , and Y. Hu . 2022. “Environmental‐Related Variation of Stoichiometric Traits in Body and Organs of Non‐Native Sailfin Catfishes *Pterygoplichthys* spp.” Ecology and Evolution 12: e9483. 10.1002/ece3.9483. 36349255 PMC9636514

[ece371920-bib-0077] Wenger, S. J. , A. L. Subalusky , and M. C. Freeman . 2019. “The Missing Dead: The Lost Role of Animal Remains in Nutrient Cycling in North American Rivers.” Food Webs 18: e00106. 10.1016/j.fooweb.2018.e00106.

[ece371920-bib-0078] Whiles, M. R. , A. D. Huryn , B. W. Taylor , and J. D. Reeve . 2009. “Influence of Handling Stress and Fasting on Estimates of Ammonium Excretion by Tadpoles and Fish: Recommendations for Designing Excretion Experiments.” Limnology and Oceanography: Methods 7: 1–7. 10.4319/lom.2009.7.1.

[ece371920-bib-0079] Wiig, E. , J. E. Reseland , K. Østbye , H. J. Haugen , and L. A. Vøllestad . 2016. “Variation in Lateral Plate Quality in Threespine Stickleback From Fresh, Brackish and Marine Water: A Micro‐Computed Tomography Study.” PLoS One 11: e0164578. 10.1371/journal.pone.0164578. 27764140 PMC5072691

[ece371920-bib-0080] Wilder, S. M. , D. Raubenheimer , and S. J. Simpson . 2016. “Moving Beyond Body Condition Indices as an Estimate of Fitness in Ecological and Evolutionary Studies.” Functional Ecology 30: 108–115. 10.1111/1365-2435.12460.

[ece371920-bib-0081] Wootton, R. 1976. The Biology of the Sticklebacks. Academic Press.

