# Supplementary material for: Investigating Stoichiometric Controls of Nutrient Recycling in Rivers Using the Threespine Stickleback (Gasterosteus aculeatus)
Source: Ecol Evol. 2025 Sep 16;15(9):e71920. doi: 10.1002/ece3.71920 (PMC12441312; doi:10.1002/ece3.71920)
Supplement: Supplementary file 3 — Data S1: Summary of site, water quality, and environmental nutrient data. [file ECE3-15-e71920-s003.docx]

**Appendix**

**Table S1**. Summary of site, water quality, and environmental nutrient data. Environmental P and N measurements are means from 3 water samples from each site. Standard deviations (SD) are reported below the means. Other environmental variables represent single measurements taken at the site on the day of sampling.

| **Site name** | **Sooke River Campground** | **Sooke River Road** | **Parking Lot 1** | **Parking Lot 3** | **Upper River Campground** |
| --- | --- | --- | --- | --- | --- |
| Abbreviation | SRC | SRR | PL1 | PL3 | URC |
| Coordinates | 48°23'23.0"N 123°42'26.0"W | 48°24'38.2"N 123°42'45.9"W | 48°25'53.4"N 123°42'54.1"W | 48°24'07.2"N 123°42'20.7"W | 48°27'48.4"N 123°44'22.8"W |
| Distance to following site (km, measured along the river) | 4.2 | 1.8 | 1.4 | 3.2 | Total river surveyed  =10.6 |
| Average temperature (°C) | 19.6 | 18.4 | 18.7 | 18.9 | 17.7 |
| Average pH | 7.52 | 6.66 | 6.71 | 7.00 | 6.83 |
| Average turbidity (NTU) | 0.19 | 0.23 | 0.21 | 0.35 | 0.09 |
| Average DO (%) | 123.2 | 99.4 | 95.2 | 102.7 | 96.7 |
| Average conductivity (mmHg) | 24987 | 62.1 | 59.5 | 59.9 | 57.6 |
| Average environmental P (µg/L) | 9.43 (SD=2.08) | 8.05  (SD=9.76) | 2.53  (SD=0.53) | 2.99  (SD=1.35) | 1.84  (SD=0.87) |
| Average environmental N (µg/L) | 5.75  (SD=2.83) | 6.79  (SD=2.28) | 8.08  (SD=2.27) | 12.71  (SD=3.69) | 7.76  (SD=3.38) |

**Table S2.** Summary statistics for morphometric measurements of sticklebacks from all sample sites (n=99). Mean, range, standard deviation (SD) and coefficient of variance (CV) are shown.

|  | **Mean** | **Range** | **SD** | **CV (%)** |
| --- | --- | --- | --- | --- |
| Standard Length (mm) | 48.71 | 36.41-68.12 | 6.33 | 13.00 |
| Body Dry Weight (g) | 0.32 | 0.137-0.688 | 0.13 | 40.98 |
| Gut Length (mm) | 27.03 | 12.94-43.22 | 5.70 | 21.08 |
| Girdle Length (mm) | 11.76 | 8.36-16.89 | 1.92 | 16.31 |
| Condition Index (%) | 100.04 | 55.53-186.75 | 22.64 | 22.63 |

**Table S3.** Summary statistics for body, gut, and excretion elemental composition. Sample size, mean, range, standard deviation (SD) and coefficient of variance (CV) are shown.

|  | **Sample size (n)** | **Mean** | **Range** | **SD** | **CV** |
| --- | --- | --- | --- | --- | --- |
| % P Body | 99 | 4.03 | 2.23-5.88 | 0.79 | 19.48 |
| % N Body | 49 | 10.05 | 6.30-15.40 | 1.65 | 16.36 |
| N:P of the body | 28 | 5.72 | 2.91-8.58 | 1.53 | 26.84 |
| %P Foregut | 93 | 1.27 | 0.36-2.48 | 0.32 | 28.39 |
| %P Hindgut | 72 | 1.46 | 0.13-3.45 | 0.32 | 23.05 |
| P Digestion ratio | 69 | 0.065 | -2.18-1.18 | 0.50 | 799.63 |
| P Excretion Rate (g/min/g dry mass) | 97 | 0.62 | 0.012-2.33 | 0.50 | 80.84 |
| N excretion rate (g/min/g dry mass) | 97 | 6.28 | 0.37-19.86 | 5.24 | 83.45 |
| N:P of excretion | 97 | 42.0 | 1.54-311.44 | 53.92 | 128.43 |

**Table S4. Summary of variation in lateral plate morphology and size between sites. Mean, range, standard deviation (SD), and coefficient of variation (CV) are shown for standard length (mm). Counts of low, partial, and fully plated morphs are shown for lateral plate morphology.**

| **Site name** |  | **Sooke River Campground (SRC)** | **Sooke River Road**  **(SRR)** | **Parking Lot 1**  **(Pl1)** | **Parking Lot 3**  **(Pl3)** | **Upper River Campground**  **(URC)** |
| --- | --- | --- | --- | --- | --- | --- |
| Standard length (mm) | mean | 55.7 | 42.5 | 47.0 | 46.9 | 50.5 |
|  | range | 50.31-62.93 | 36.41-52.4 | 41.58-68.12 | 37.27-52.35 | 33.79-67.54 |
|  | SD | 3.11 | 3.16 | 5.22 | 3.92 | 5.79 |
|  | CV | 5.58 | 7.44 | 11.1 | 8.35 | 11.4 |
| Lateral plate morphology (count) | Low | 0 | 1 | 6 | 11 | 22 |
|  | Partial | 0 | 0 | 7 | 0 | 0 |
|  | Full | 22 | 21 | 9 | 0 | 0 |

**Table S5.** Summary of global models for body, gut, and excretion. All global and top models for excretion include site as a random effect.

| **Response** | **Global Model** |
| --- | --- |
| % P Body | ~ sex + plate morph + condition index |
| Ln(Body N:P) | ~ sex + plate morph + condition index |
| Foregut %P | ~ sex + plate morph + gut length |
| P digestion ratio | ~ sex + plate morph + gut length |
| Hindgut %P | ~ sex + plate morph + gut length |
| Ln(P excretion rate) | ~ sex + body %P + standard length |
| Ln(N:P excretion) | ~ sex + body N:P + standard length |

**Table S6.** Model selection table for body %P. The global model predictors include plate morph, condition index, sex, and site as a random factor. The + indicates that a variable was included in the model.

| **Body %P top models** | | | **Coefficients** | | | |
| --- | --- | --- | --- | --- | --- | --- |
| **Rank** | **ΔAICc** | **Weight** | **Intercept** | **Plate morph** | **Condition index** | **Sex** |
| 1 | 0 | 0.76 | 6.09 | + | -0.017 | - |
| 2 | 3.80 | 0.11 | 5.81 | - | -0.018 | - |
| 3 | 4.03 | 0.10 | 5.50 | + | -0.019 | + |

**Table S7.** Model selection table for log transformed body N:P. The global model predictors include plate morph, condition index, sex, and site as a random factor.

| **Body N:P top models** | | | **Coefficients** | | | |
| --- | --- | --- | --- | --- | --- | --- |
| **Rank** | **ΔAICc** | **Weight** | **Intercept** | **Plate morph** | **Condition index** | **Sex** |
| 1 | 0 | 0.838 | 1.71 | - | - | - |
| 2 | 4.5 | 0.088 | 1.85 | + | - | - |

**Table S8.** Top GLMMs for log transformed P excretion rate. Model predictors include standard length and body %P.

| **Ln(P excretion rate) top models** | | | **Coefficients** | | |
| --- | --- | --- | --- | --- | --- |
| **Rank** | **ΔAICc** | **Weight** | **Intercept** | **Body %P** | **Standard Length** |
| 1 | 0 | 0.69 | -5.37 | - | 0.067 |
| 2 | 1.83 | 0.28 | -5.75 | 0.18 | 0.060 |

**Table S9.** Top GLMMs for log transformed N:P of excretion. Model predictors are standard length, sex, body N:P, and site as a random factor.

| **Ln(N:P excretion rate) top models** | | | **Coefficients** | | | |
| --- | --- | --- | --- | --- | --- | --- |
| **Rank** | **ΔAICc** | **Weight** | **Intercept** | **Body N:P** | **Standard Length** | **Sex** |
| 1 | 0 | 0.67 | 2.82 | - | - | - |
| 2 | 2.71 | 0.17 | 2.50 | 0.18 | - | - |
| 3 | 4.07 | 0.088 | 2.86 | - | - | + |

**Table S10.** Top GLMMs for log transformed P excretion rate including foregut %P. Model predictors are standard length, sex, foregut %P, body %P, and site as a random factor.

| **Ln(P excretion rate) top models** | | | **Coefficients** | | | | |
| --- | --- | --- | --- | --- | --- | --- | --- |
| **Rank** | **ΔAICc** | **Weight** | **Intercept** | **Body %P** | **Standard Length** | **Sex** | **Foregut %P** |
| 1 | 0 | 0.38 | -6.07 | 0.22 | 0.064 | - | - |
| 2 | 0.32 | 0.33 | -5.75 | - | 0.071 | - | - |
| 3 | 2.94 | 0.088 | -5.39 | - | 0.073 | - | -0.2 |
| 4 | 3.01 | 0.085 | -5.92 | 0.21 | 0.066 | - | -0.17 |

**Table S11.** Full average of P excretion model. Model predictors include body %P, standard length, and foregut %P.

|  | **Estimate** | **SD** | **P value** |
| --- | --- | --- | --- |
| Intercept | -5.80 | 0.81 | <0.001 |
| Body %P | 0.11 | 0.13 | 0.367 |
| Standard Length | 0.068 | 0.014 | <0.001 |
| Foregut %P | -0.036 | 0.11 | 0.745 |

**Table S12.** Model selection table for log transformed excretion N:P including foregut N:P. The global model predictors include body N:P, foregut N:P, plate morph, standard length, sex, and site as a random factor.

| **Top models for N:P of excretion** | | | **Coefficients** | | | | | |
| --- | --- | --- | --- | --- | --- | --- | --- | --- |
| **Rank** | **ΔAICc** | **Weight** | **Intercept** | **Body N:P** | **Foregut N:P** | **Plate morph** | **Standard length** | **Sex** |
| 1 | 0 | 0.53 | 2.82 | - | - | - | - | - |
| 2 | 2.71 | 0.14 | 2.50 | 0.18 | - | - | - | - |
| 3 | 3.06 | 0.12 | 2.78 | - | 0.013 | - | - | - |
| 4 | 4.07 | 0.069 | 2.86 | - | - | - | - | + |

**Table S13.** Full average of N:P excretion model. Model predictors include body N:P, foregut N:P and site as a random factor.

|  | **Estimate** | **SD** | **P value** |
| --- | --- | --- | --- |
| Intercept | 2.76 | 0.73 | <0.001 |
| Body N:P | 0.032 | 0.17 | 0.85 |
| Foregut N:P | 0.0018 | 0.14 | 0.99 |
